# Design, purification and assessment of GRP78 binding peptide-linked Subunit A of Subtilase cytotoxic for targeting cancer cells

**DOI:** 10.1186/s12896-016-0294-5

**Published:** 2016-09-01

**Authors:** Lichao Zhang, Zongwei Li, Tonglin Shi, Xiaoqin La, Hanqing Li, Zhuoyu Li

**Affiliations:** 1 Institute of Biotechnology, Key Laboratory of Chemical Biology and Molecular Engineering of National Ministry of Education, Shanxi University, Taiyuan, 030006 China; 2 School of Life Science, Shanxi University, Taiyuan, 030006 China; 3 College of Life Science, Zhejiang Chinese Medical University, Hangzhou, 310053 China

**Keywords:** Glucose-regulated protein 78, GRP78 binding peptide, Subunit A of Subtilase cytotoxic, Cancer, Apoptosis

## Abstract

**Background:**

Targeted therapies for cancer, especially the malignant cancer, are always restricted by the deficiency of tumor-specific drug delivery methods. Subtilase cytotoxic is a virulent cytotoxin, and the subunit A (SubA) of it is able to destroy the structure of glucose-regulated protein 78 (GRP78) to induce cell apoptosis, and to be expected as anti-cancer drugs, however, the ubiquitous receptor of subunit B of Subtilase cytotoxic (SubB) restricts its application on cancer therapy.

**Results:**

The present study constructed and expressed a fusion protein of GBP-SubA in *E. coli* Rosetta (DE3) system, in which the subunit B of Subtilase cytotoxic was replaced by GRP78 binding peptide (GBP). The fusion protein was expressed in inclusion body form. Subsequently, the denaturation/renaturation process and Ni-column purification were performed. Our data indicated the purified GBP-SubA could bind GRP78 existed on cancer cell surface specifically, internalize into cells to inactivate intracellular GRP78 and induce apoptosis. Moreover, the apoptosis induction effect of GBP-SubA was enhanced obviously along with the increased cancer cell surface GBP78.

**Conclusions:**

It indicates that the recombinant GBP-SubA possesses the dual functions of GBP and SubA to induce cancer cell apoptosis specifically, revealing that GBP-SubA holds important implications for developing as an anti-cancer peptide drug.

**Graphical abstract:**

A schematic representation of the construction and function of GBP-SubA. 
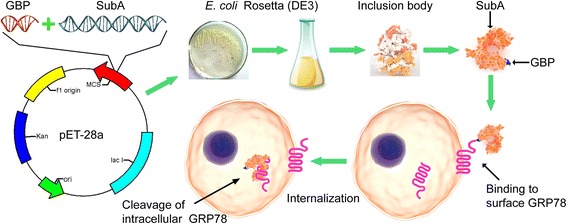

**Electronic supplementary material:**

The online version of this article (doi:10.1186/s12896-016-0294-5) contains supplementary material, which is available to authorized users.

## Background

Glucose-regulated protein 78 (GRP78), also known as the immunoglobulin heavy chain binding protein (BiP), is a member of the heat-shock chaperon family that mainly resides in endoplasmic reticulum (ER) [1]. In normal cells, GRP78 is considered as an important regulator of ER homeostasis because of its roles in protein folding and assembly, targeting misfolded proteins for degradation, controlling the activation of the ER transmembrane sensor proteins and maintenance of Ca^2+^ homeostasis [2]. Accumulated reports have shown the cancer cells usually suffer hypoxia, acidosis and glucose deprivation, which often induce ER stress and GRP78 high expression [3, 4].

The excessive GRP78 expression is associated with tumor cell apoptosis resistance [5], drug resistance [6], metastasis [7] and metabolic reprogramming [8]. Decreasing the expression or disabling the function of GRP78 can promote the apoptosis and inhibit tumor growth [9]. More importantly, the cancer cell surface GRP78 is increased along with stressful conditions [10, 11]. In addition, the cell surface GRP78 often presents in the form of clustering or punctate aggregates that deals with carcinogenesis [12]. All of these characters suggest that the GRP78 is capable to serve as a receptor and target of antitumor drugs.

Current antitumor chemotherapeutic drug is often limited by severe side effects that leave patients under extreme distress. To increase the delivery efficiency and decrease undesirable side effects, it is essential to develop new method that facilitate the cancer cell-specific delivery of therapeutic agents. Recently, peptide ligands have gained increasing attention for their specific cell targeting and the delivery of payloads into the target cells [13, 14]. GRP78 binding peptide (GBP: WIFPWIQL) consisted of eight amino acids was designed by Marco and his colleagues, which was able to specifically bind to tumor cell surface GRP78, and deliver the fused pro-apoptotic protein into the tumor cells to suppress tumor growth [15]. Therefore, GBP is very suitable as the vehicle of cytotoxic agents to suppress tumor growth with fewer side effects.

Subtilase cytotoxic is the most recently discovered in the Shiga toxigenic *Escherichia coli* (STEC) O113:H21 strain 98NK2, and it is a member of AB_5_ toxins family [16, 17]. The Subtilase cytotoxic holotoxin is composed of one 35 kD catalytic A subunit (SubA) and five 13 kD B subunits (SubB). SubB can bind to glycan receptors (Neu5Gc) that universally exist on mammalian cell surface, and SubB is necessary for internalization of the holotoxin. SubA is the catalytic subunit, its serine protease activity is responsible for toxicity to the host cells [18]. Moreover, SubA possesses the extreme substrate specificity. The analysis from proteomics and functional studies reveals that GRP78 is the specific molecular target for SubA. It cleaves GRP78 between the amino acid residues Leu^416^ and Leu^417^ that locate within the hinge region between the ATPase and COOH-terminal protein binding domains [19]. The cleavage at this site leads to loss of GRP78 function and exerts fatal consequences for the cells [20].

Here, a fusion protein GBP-SubA was constructed and obtained from inclusion bodies through denaturation and renaturation process. The experiment confirmed that the fusion protein kept the native features of GBP and SubA simultaneously. It possessed dual efficacy of targeting and killing tumor cells by against GRP78 only, but with less effect on normal cells. This study may provide a new strategy for developing targeted anti-tumor drugs.

## Methods

### Reagents

Plasmid pET-28a was preserved in our laboratory. *Taq* DNA polymerase, DNA Ligation Kit, and restriction enzymes were obtained from Takara Biotech Co., Ltd. (Dalian, China). The Plasmid Mini Kit and Gel Extraction Kit were purchased from Omega (Norcross, USA). RPMI-1640 medium and DMEM/F12 (1:1) medium were from Hyclone (Logan, USA). Fetal bovine serum (FBS) was from Sangon biotech (Shanghai, China). Antibodies for His-tag and GRP78 were purchased from Proteintech (Wuhan, China). Antibody for GRP78 N-terminal was from Beyotime Biotechnology (Shanghai, China). Phycoerythrin-conjugated secondary antibody was obtained from Santa Cruz Biotechnology (Dallas, USA). Rhodamine Phalloidin was purchased from Cytoskeleton (Denver, USA). Guava Nexin Reagent and polyvinylidene difluoride (PVDF) membrane were from Millipore (Darmstadt, Germany). BCA protein assay kit and Immunol Staining Fix Solution were from Beyotime (Jiangsu, China). Enhanced chemiluminescence detection kit was from Engreen (Beijing, China). All other chemicals and reagents were obtained from Sigma (St. Louis, USA).

### Cell lines and strains

Human cell lines DLD1, HepG2 and HL-7702 were obtained from the Cell Bank of the Chinese Academy of Sciences (Shanghai, China). DLD1 and HepG2 cells were grown at 37 °C in RPMI-1640 medium supplemented with 10 % heat inactivated FBS, 50 IU penicillin and 50 μg/mL streptomycin. HL-7702 cells were grown at 37 °C in DMEM/F12 (1:1) medium supplemented with 10 % heat inactivated FBS, 50 IU penicillin and 50 μg/mL streptomycin. The *Escherichia coli* strains DH5α, BL21 (DE3) and Rosetta (DE3) were preserved in our laboratory and stored in Luria-Bertani (LB) medium containing 15 % glycerol at −80 °C.

### Recombinant plasmid construction

The DNA encoding GBP (WIFPWIQL) and SubA (Gene ID: 3654564) were fused and synthesized by TaKaRa Biotechnology (Dalian, China), and the restriction sites of *Bam* HI and *Xho* I were separately introduced to 5′ and 3′ ends of the fused DNA. The synthesized GBP-SubA DNA segment was ligated into T-Vector pMD19 (TaKaRa, Dalian, China). The recombinant plasmid pMD19-GBP-SubA and plasmid pET-28a were digested using *Bam* HI and *Xho* I in buffer K at 30 °C for 2 h. After gel extraction and purification, GBP-SubA DNA segment was ligated into pET-28a vector using DNA Ligation Kit with a ratio of insert: vector = 5:1 (mol/mol) as the user manual. Recombinant pET-28a-GBP-SubA was transformed into *E. coli* Rosetta (DE3) cells. Cells were grown overnight at 37 °C on LB plates with kanamycin. Positive colonies were identified by colony PCR and restriction digestion, and verified by DNA sequencing (Sangon, Shanghai, China).

### Expression of the recombinant protein

Six histidine-tagged fusion protein GBP-SubA was expressed in the host strain *E. coli* Rosetta (DE3) cells. Briefly, *E. coli* Rosetta (DE3) cells containing pET-28a-GBP-SubA were streaked on a LB-agar plate containing 50 μg/mL kanamycin and incubated overnight at 37 °C. A single colony from the plate was picked and inoculated into 15 mL LB-broth supplemented with 50 μg/mL kanamycin and grown at 37 °C overnight. The 15 mL bacterial solution was inoculated into 2 L LB medium containing 50 μg/mL kanamycin in a shaker at 37 °C until the A_600_ of culture reached to 0.8–0.9. Following induction with 1 mM isopropyl-β-D-thiogalactopyranoside (IPTG) for 4 h at 37 °C with 200 rpm shaking, cells were harvested by centrifugation at 8, 000 g, 4 °C for 20 min and washed with cold phosphate buffered saline (PBS, 8 g/L NaCl, 3.63 g/L Na_2_HPO_4_•12 H_2_O, 0.2 g/L KCl and 0.24 g/L KH_2_PO_4_, pH = 8.0). The pellets were resuspended in lysis buffer (PBS with 5 mM β-mercaptoethanol, 1 % (w/v) sodium deoxycholate and 1 mM PMSF, pH = 8.0) at a ratio of 1:5 (w/v) and lysed using ultrasound. The sonicated cell suspensions were isolated by centrifugation at 12, 000 g and 4 °C for 30 min, and the supernatant and pellet were applied to 10 % sodium dodecyl sulfate polyacrylamide gel electrophoresis (SDS-PAGE) to determine the recombinant protein solubility and expression level. Gels were scanned using Canon CanoScan 9000F Mark II scanner. The absolute integrated optical density of each band was quantitated using GelPro Analyzer software from Media Cybernetics (Des Moines, IA). The ratio of target protein to the total proteins represented the enrichment of GBP-SubA fusion protein.

### GBP-SubA purification and refolding

The insoluble fraction containing GBP-SubA was washed three times in wash buffer (PBS with 4 M urea and 1 % Triton X-100, pH = 8.0) to remove as much contaminants as possible. Then, the insoluble fraction containing GBP-SubA was resuspended in extraction buffer (PBS with 6 M guanidine-HCl, 0.5 M NaCl, 20 mM imidazole and 5 mM β-mercaptoethanol, pH = 8.0) and dissolved overnight at 4 °C. The dissolved solution of precipitate was centrifuged at 12, 000 g for 30 min to remove any insoluble debris. The supernatant was mixed with Ni Sepharose 6 Fast Flow (GE healthcare) which had been pre-equilibrated with extraction buffer and shaken gently overnight at 4 °C. The mixture was loaded on a PD-10 column (GE healthcare). The column was then washed with 40 mL extraction buffer, followed by 30 mL elution buffer A (PBS with 8 M urea, 0.5 M NaCl, 20 mM imidazole and 5 mM β-mercaptoethanol, pH = 8.0), and then 20 mL elution buffer B (PBS with 8 M urea, 0.5 M NaCl, 20 mM imidazole and 5 mM β-mercaptoethanol, pH = 6.3). The GBP-SubA was then eluted with a gradient of 0–500 mM imidazole in elution buffer B. The eluted protein solutions were collected and analyzed by SDS-PAGE.

The purified GBP-SubA was dialyzed at 4 °C. Briefly, the denatured GBP-SubA was refolded by dialysis against 200 mL elution buffer A to 800 mL of refolding buffer (PBS with 0.1 mM glutathione, 0.01 mM glutathione disulfide, 1 mM EDTA, 0.15 M L-arginine and 5 % (v/v) glycerol, pH = 8.0), which was added dropwise at a rate of 100 mL/h. This was followed by dialysis against three changes of refolding buffer. At last, the GBP-SubA solution dialysised against 2 L PBS. The purity of GBP-SubA was judged by SDS-PAGE after staining with coomassie brilliant blue R-250 and AgNO_3_. Concentration of refolded GBP-SubA was determined by BCA protein assay kit (Beyotime, Jiangsu, China).

### Flow cytometric assay of cell surface GRP78 and cell apoptosis

The expression of GRP78 on cell surfaces was evaluated by flow cytometry. HL-7702, DLD1 and HepG2 cells were collected using cell scrapers after double washes in PBS, and then cells were pipetted dozens to make single-cell suspension. After centrifugation at 300 g for 5 min at room temperature, cells were incubated with anti-GRP78 primary antibodies and sequentially phycoerythrin-conjugated secondary antibodies. Each of antibodies was incubated at 37 °C for 1 h in dark, and gently mixed cells every 10 min during this procedure. Cells were washed triple and loaded onto a Guava PCA flow cytometer (Millipore, USA) to measure, at least 20, 000 cells were counted per sample, and data were analyzed and exported by CytoSoft 6.0.2.

The apoptosis of cells with GBP-SubA treatment was evaluated by flow cytometry. HL-7702, DLD1 and HepG2 cells were added to 60-mm culture plates at a density of 10^6^ cells per dish and incubated overnight at 37 °C in 5 % CO_2_. The purified 0.1 μg/mL GBP-SubA was added to each dish and incubated for 48 h. In the GRP78-blocking assay, the antibody of GRP78 was add in the cell medium to incubate for 1 h, and then added GBP-SubA. At last, the cells were harvested and operated according the instruction of Guava Nexin Reagent.

### Western blotting

For western blotting, after appropriate treatments, lysates of cells were prepared and centrifuged at 13, 000 g at 4 °C to remove cell debris. 80 μg of the obtained supernatant proteins were mixed with 5× SDS sample buffer, and boiled for 5 min. After the samples had been separated, the proteins on the gel were transferred onto a PVDF membrane. After blocking in 5 % skim milk for 1 h, the membranes were incubated overnight at 4 °C with the appropriate diluted primary antibodies, followed by incubation with HRP-conjugated secondary antibody at 37 °C for 2 h. The bands were visualized using an enhanced chemiluminescence detection kit and radiographic film exposure. Documentation of blots was performed by Canon CanoScan 9000F Mark II scanner.

### Immunofluorescence analysis of cytoplasmic GBP-SubA

In the process of cytoplasmic GBP-SubA staining, HL-7702, DLD1 and HepG2 Cells were plated on 12-well glass slides, and treated with 0.1 μg/mL GBP-SubA for 12 h. In the GRP78-blocking assay, the antibody of GRP78 was add in the cell medium to incubate for 1 h, and then added GBP-SubA. After treatments, the cells were fixed in Immunol Staining Fix Solution overnight at 4 °C, and permeabilized in PBS containing 0.3 % Triton X-100 for 10 min. Next, slides were blocked in PBS containing 5 % bovine serum albumin (BSA) for 30 min at 37 °C, and incubated with the anti-His-tag antibodies at 4 °C overnight. Slides were then washed and incubated with corresponding FITC-conjugated secondary antibodies and Rhodamine-conjugated Phalloidin. Nucleus was identified by staining with DAPI. After three washes of PBS, slides were mounted in gelvatol for immunofluorescence analysis. All images were obtained by DeltaVision Personal microscope (Applied Precision Inc.).

### Cell proliferation assay

For MTT assay, HL-7702, DLD1 and HepG2 cells were added to 96-well culture plates at a density of 10^4^ cells per well and incubated overnight at 37 °C in 5 % CO_2_. The purified recombinant GBP-SubA in different concentrations were added to each well, and PBS was used as the control. In the GRP78-blocking assay, before the 0.1 μg/mL GBP-SubA was added in the medium, the antibody of GRP78 was added and incubated for 1 h. After 48 h incubation, 20 μL (final concentration is 0.5 mg/mL) 3-(4,5-dimethylthiazol-2-yl)-2,5-diphenyltetrazolium bromide (MTT) was added to each well and incubated for 4 h at 37 °C. The solution was discarded and 100 μL DMSO was added to each well. After shaking gently for 10 min, the plate was read at 570 nm using TECAN infinite M200 Pro microplate spectrophotometer.

For cytometry assay, HL-7702, DLD1 and HepG2 cells were added to 12-well culture plates at a density of 5 × 10^4^ cells per well and incubated overnight at 37 °C in 5 % CO_2_. Then 0.1 μg/mL GBP-SubA was added to the well, and PBS was used as the control. After 48 h of incubation, the cells were collected by trypsinization and counted with hemacytometer.

### TUNEL and DAPI staining

TUNEL staining data were obtained using a commercial kit (Cat. No. KGA7071, KeyGEN BioTECH, Nanjing, China). Briefly, HL-7702, DLD1 and HepG2 Cells were plated on 12-well glass slides. After treatments with 0.1 μg/mL GBP-SubA for 48 h, the cells were fixed in 4 % paraformaldehyde for 30 min, and permeated with Proteinase K solution for 30 min. Then slides were incubated in TUNEL reaction mixture containing terminal deoxynucleotidyl transferase (TDT) for 60 min at 37 °C. The slides were then washed in PBS for three times, counterstained with 4′,6-diamidino-2-phenylindole (DAPI) for 10 min, and rinsed with PBS. The sections were analyzed under a fluorescent microscope. In the GRP78-blocking assay, before the 0.1 μg/mL GBP-SubA was added in the medium, the antibody of GRP78 was added and incubated for 1 h.

### Statistical analysis

Data were expressed as the mean ± SEM. Differences among groups were tested by one-way analysis of variance (ANOVA). Comparisons between two groups were evaluated using Student’s *t*-test. A value of *p* < 0.05 was considered statistically significant.

## Results

### Plasmid construction and expression of GBP-SubA

**Fig. 1:**
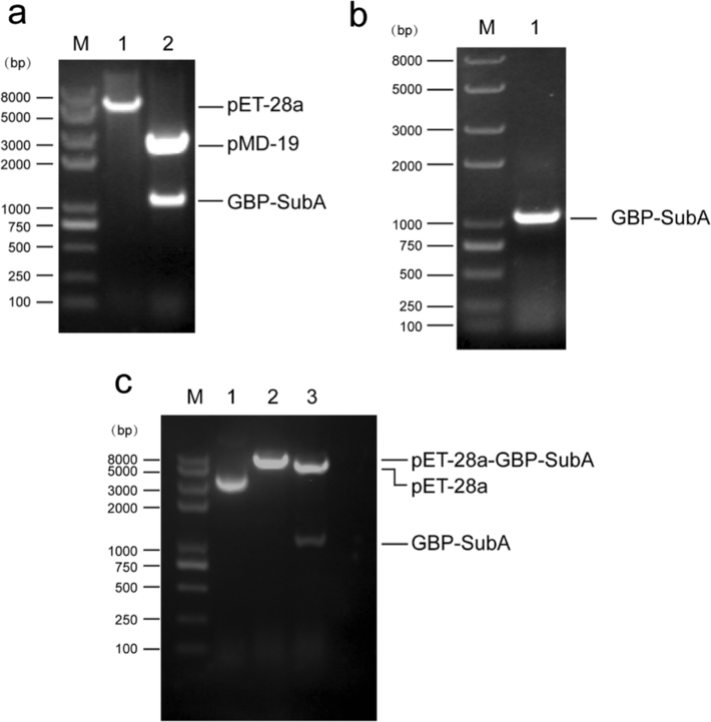
Construction of the recombinant plasmid pET-28a-GBP-SubA. **a**. Both pET-28a and pMD19-GBP-SubA plasmids were digested by *Bam* HI and *Xho* I. Lane 1: pET-28a vector (after digestion); lane 2: pMD19-GBP-SubA plasmid (after digestion). **b**. GBP-SubA was successfully inserted into pET-28a vector confirmed by colony PCR. Lane 1: PCR products amplified by GBP-SubA primers. **c**. Both pET-28a and recombinant pET-28a-GBP-SubA were digested by *Bam* HI or/and *Xho* I. Lane 1: pET-28a vector (before digestion); lane 2: recombinant pET-28a-GBP-SubA (digested with *Bam* HI only); lane 3: recombinant pET-28a-GBP-SubA (digested with *Bam* HI and *Xho* I); Lane M: marker

To get the gene of SubA, we commissioned TaKaRa Biotechnology Company to synthesize and insert it into T-Vector pMD19. The synthesized DNA was 1089 bp and encoded an ORF of 355 amino acid residues that included eight amino acids of GBP. Subsequently, the pMD19-GBP-SubA and pET-28a vector were digested by restriction enzyme to release the GBP-SubA insert and prepare the vector for ligation and transformation (Fig. 1a). The recombinant plasmid pET-28a-GBP-SubA was successfully constructed and confirmed by colony PCR (Fig. 1b, the primers were listed in Additional file 1: Table S1), restriction digestion (Fig. 1c) and DNA sequencing.

**Fig. 2:**
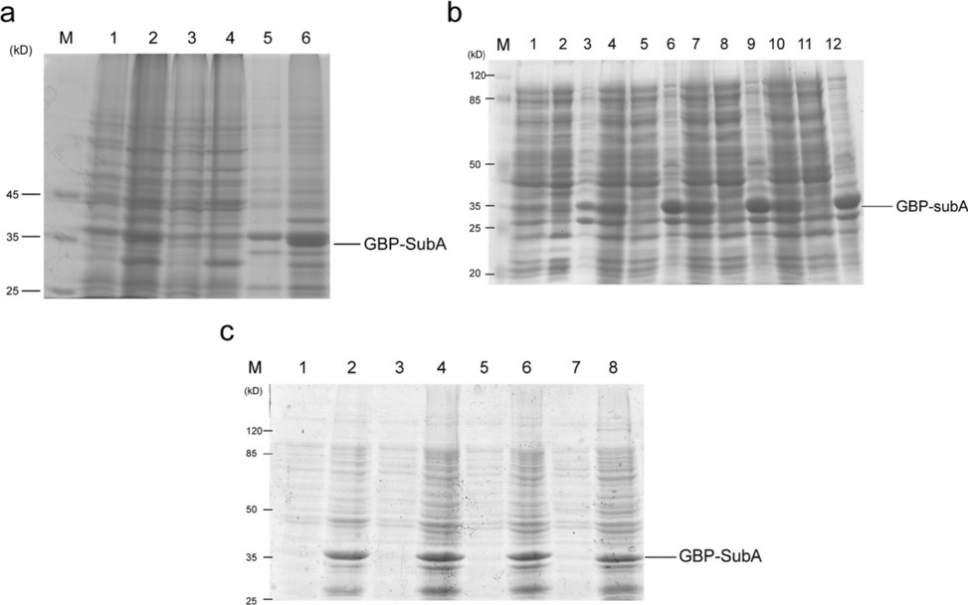
Expression of GBP-SubA. **a**. *E. coli* Rosetta (DE3) containing pET-28a or pET-28a-GBP-SubA was induced by 1 mM IPTG at 37 °C for 4 h, and then lysed with ultrasound. Lane 1, 3 and 5: the total cellular lysate, soluble lysate fraction and insoluble lysate fraction from *E. coli* Rosetta (DE3) containing pET-28a plasmid, respectively; lane 2, 4 and 6: total cellular lysate, soluble lysate fraction and insoluble lysate fraction from *E. coli* Rosetta (DE3) containing pET-28a-GBP-SubA plasmid, respectively; Lane M: marker. **b**. Induction of GBP-SubA with different concentrations of IPTG at 37 °C. lane 1–3: total cellular lysate, soluble lysate fraction and insoluble lysate fraction of *E. coli* Rosetta (DE3) containing pET-28a-GBP-SubA plasmid that induced by 0.1 mM IPTG; lane 4–6: total cellular lysate, soluble lysate fraction and insoluble lysate fraction of *E. coli* Rosetta (DE3) containing pET-28a-GBP-SubA plasmid that induced by 0.5 mM IPTG; lane 7–9: total cellular lysate, soluble lysate fraction and insoluble lysate fraction of *E. coli* Rosetta (DE3) containing pET-28a-GBP-SubA plasmid that induced by 1 mM IPTG; lane 10–12: total cellular lysate, soluble lysate fraction and insoluble lysate fraction of *E. coli* Rosetta (DE3) containing pET-28a-GBP-SubA plasmid that induced by 2 mM IPTG; Lane M: marker. **c**. Induction of GBP-SubA with different concentrations of IPTG at 16 °C. Lane 1–2: soluble lysate fraction and insoluble lysate fraction of *E. coli* Rosetta (DE3) containing pET-28a-GBP-SubA plasmid that induced by 0.1 mM IPTG; lane 3–4: soluble lysate fraction and insoluble lysate fraction of *E. coli* Rosetta (DE3) containing pET-28a-GBP-SubA plasmid that induced by 0.5 mM IPTG; lane 5–6: soluble lysate fraction and insoluble lysate fraction of *E. coli* Rosetta (DE3) containing pET-28a-GBP-SubA plasmid that induced by 1 mM IPTG; lane 7–8: soluble lysate fraction and insoluble lysate fraction of *E. coli* Rosetta (DE3) containing pET-28a-GBP-SubA plasmid that induced by 2 mM IPTG; Lane M: marker

To obtain the fusion proteins of GBP-SubA, we transfected the recombinant plasmid pET-28a-GBP-SubA into *E. coli* Rosetta (DE3) cells, and the cells were induced by 1 mM IPTG at 37 °C for 4 h. However, most of target proteins were found in the insoluble fraction after the centrifugation following the cell lysis (Fig. 2a). Several other expression parameters, including IPTG concentrations and incubation temperatures, were assessed, but none of them can increase the soluble or insoluble expression of GBP-SubA (Fig. 2b and c). So the above expression condition was chosen to obtain inclusion bodies, and the denaturation and renaturation were followed.

### Purification of GBP-SubA

**Fig. 3:**
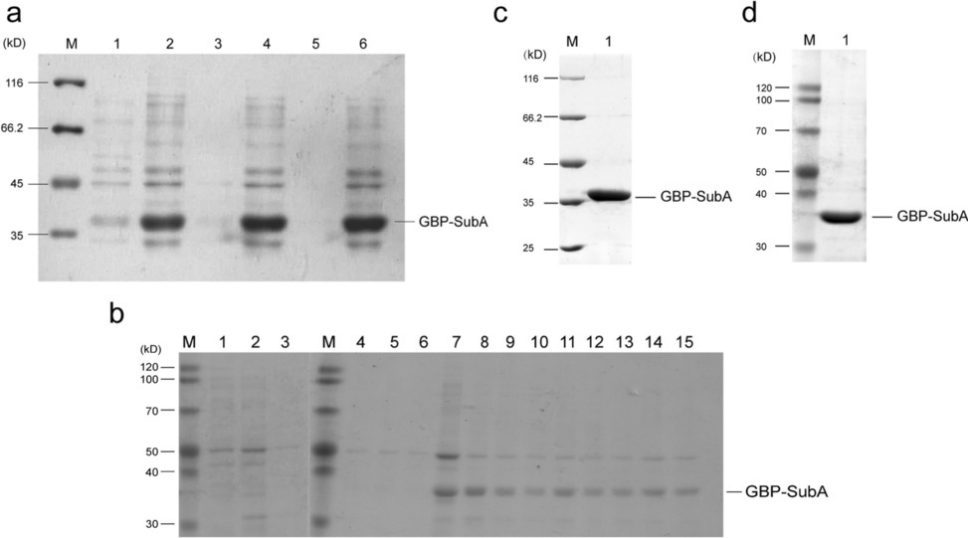
Purification and refolding of GBP-SubA. **a**. After three washes with PBS, the irrelevant proteins of insoluble lysate fraction reduced. Lane 1 and 2: supernatant and pellet after the 1^st^ wash; lane 3 and 4: supernatant and pellet after the 2^nd^ wash; lane 5 and 6: the supernatant and pellet after the 3rd wash; Lane M: marker. **b**. GBP-SubA proteins were eluted by a gradient concentration of imidazole from nickel ion-chelated beads. Lane 1–3: the protein eluted by 50 mM imidazole; lane 4–6: the protein eluted by 100 mM imidazole; Lane 7–9: the protein eluted by 200 mM imidazole; Lane 10–12: the protein eluted by 300 mM imidazole; Lane 13–15: the protein eluted by 500 mM imidazole; Lane M: marker. **c**. Refolded-GBP-SubA protein was analyzed by SDS-PAGE and coomassie brilliant blue R-250 stain. Lane 1: the GBP-SubA protein after refolding; Lane M: marker. **d**. Refolded-GBP-SubA protein was analyzed by SDS-PAGE and silver stain. Lane 1: the GBP-SubA protein after refolding; Lane M: marker

Next, the collected inclusion bodies were purified by wash buffer (containing 4 M urea). The results showed that irrelevant proteins were reduced significantly after three washes, and the loss from above procedure on GBP-SubA inclusion bodies was quite small (Fig. 3a). After dissolution with extraction buffer (containing 6 M guanidine-HCl) overnight at 4 °C, the GBP-SubA solution was loaded onto Ni-NTA resin and a series of elution buffers (contain 8 M urea and gradient imidazole concentrations) were used to elute this protein. The results showed that GBP-SubA was mainly eluted by elution buffer containing 200 mM imidazole, a few proteins were found in the 300 and 500 mM imidazole elution buffer, so the GBP-SubA in 200, 300 and 500 mM imidazole elution buffer were collected for the further renaturation process (Fig. 3b).

The denatured GBP-SubA was refolded by dialysis against elution buffer (without imidazole) to which PBS was added dropwise. After the concentration of urea reached to 0.01 M, the refolding buffer was replaced by PBS and continued to dialyze 12 h, at last, the yield of the refolded proteins was 3.5 mg of 1 L culture. The purity of the refolded proteins in PBS was more than 99 %, which was determined by coomassie blue stain and silver stain after SDS-PAGE (Fig. 3c and d).

### The dual bioactivity assays of the recombinant fusion proteins

**Fig. 4:**
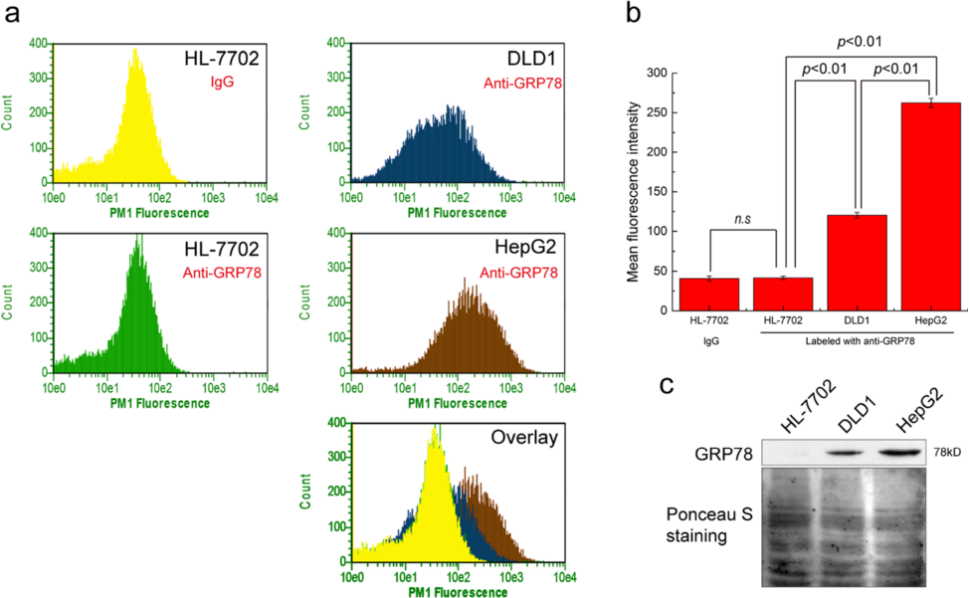
Detection of cell surface GRP78. **a**. The surface-GRP78 of HL-7702, DLD1 and HepG2 cells were stained by anti-GRP78 antibody and analyzed by flow cytometry, and the HL-7702 stained with IgG served as the negative control. **b**. The mean fluorescence intensity of HL-7702, DLD1 and HepG2 cells stained with anti-GRP78 antibody and the negative control stained with IgG. **c**. Cell membrane proteins were extracted from HL-7702, DLD1 and HepG2 cells. Equal amount of protein was loaded per lane for western blotting detection of GRP78

The best way to examine the dual bioactivities of recombinant GBP-SubA is to detect whether the GBP-SubA can enter into the cells with surface-GRP78, and whether the entered GBP-SubA can cleave the intracellular GRP78. In order to achieve these, two tumor cell lines (DLD1 and HepG2) and one normal cell line (HL-7702) were selected by flow cytometry firstly. As shown in Fig. 4a and b, tumor cell (DLD1 and HepG2) surface GRP78 is more than the normal cells, and more GRP78 were detected on HepG2 cells surface than that on DLD1. Moreover, the fluorescence intensity of HL-7702 cells was similar with the negative control, suggesting that the GRP78 was almost nonexistent on HL-7702 cell surface. Next, the total membrane proteins of the three cell lines were extracted and subjected to western blotting analysis. As shown in Fig. 4c, in the case of equal protein loading, surface GRP78 level of HepG2 cells was higher than that of DLD1 cells, and the GRP78 on HL-7702 cell surfaces was barely detectable. So the HL-7702, DLD1 and HepG2 were used as typical cells without cell surface GRP78, with little cell surface GRP78 and with a lot of cell surface GRP78, respectively.

**Fig. 5:**
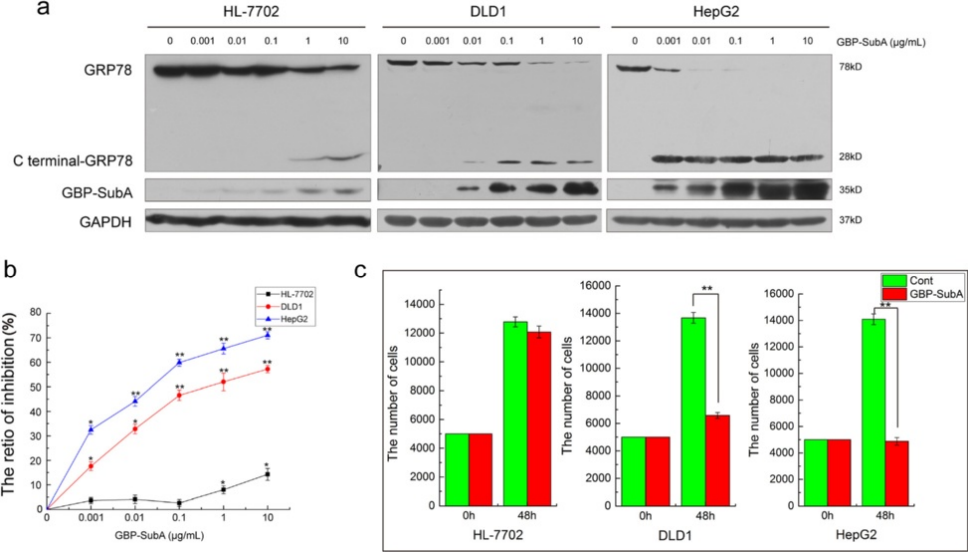
The cleavage of GRP78 and inhibition of tumor cells by GBP-SubA. **a**. The total cellular lysate was extracted from HL-7702, DLD1 and HepG2 cells that were treated with different concentrations of GBP-SubA for 24 h, and the cleavage of GRP78 was analyzed by western blotting. **b**. Cell inhibition ratios of GBP-SubA were determined by the MTT assay. HL-7702, DLD1 and HepG2 cells were incubated with different concentrations of GBP-SubA for 48 h. *p < 0.05, **p < 0.01, compared with the control. **c**. The cellular inhibition of GBP-SubA was determined by cytometry. HL-7702, DLD1 and HepG2 cells were incubated with 0.1 μg/mL GBP-SubA for 48 h. *p < 0.05, **p < 0.01, compared with the control

To analyze the cellular entry of GBP-SubA and the cleavage of GRP78, different doses of GBP-SubA were applied to above three cell lines in culture for 24 h. The total proteins of cells were extracted and analyzed by western blot. As shown in Fig. 5a, a number of GBP-SubA were detected by His-tag antibodies in HepG2 cells even at the minimum concentration of 0.001 μg/mL, the GBP-SubA in DLD1 cells also showed a dose-dependent increase. However, only a little GBP-SubA entered HL-7702 cells even at the concentration of 1 μg/mL. Simultaneously, the cleavage of GRP78 was analyzed by polyclonal anti-GRP78 antibodies. The GRP78 antibody labelled a 78 kD band in the lysates of untreated cell. By contrast, the lysate of cells treated with GBP-SubA had a markedly diminished 78 kD band, and a new band occurred at 28 kD. Along with the increase of intracellular GBP-SubA, GRP78 was cleaved in DLD1 and HepG2 cells more and more significantly, whereas the GRP78 was just slightly cleaved even at the concentration of 1 μg/mL in HL-7702 cells (Fig. 5a).

In order to evaluate the inhibition effect of GBP-SubA on tumor cells, MTT assays were performed with HL-7702, DLD1 and HepG2 cells. As shown in Fig. 5b, GBP-SubA could suppress the proliferation of HepG2 and DLD1 cells but not for HL-7702 cells. At the concentration of 0.1 μg/mL, the inhibition rates of GBP-SubA on HepG2 and DLD1 were 60 % and 47 %, respectively, whereas the HL-7702 cells were not affected conspicuously. In addition, the cytometry was applied to further verify the inhibition effect of 0.1 μg/mL GBP-SubA on tumor cells. The results revealed that the proliferation of tumor cells were specifically inhibited with 0.1 μg/mL GBP-SubA treatment, and the normal cells were not affected (Fig. 5c). These results suggested that the SubA and GBP retained their bioactivity after fusion and renaturation. 0.1 μg/mL was the suitable dose for targeting antitumor activity of GBP-SubA. So the concentration of 0.1 μg/mL of GBP-SubA was selected to perform the following experiments.

### Apoptosis-induction effect of GBP-SubA on tumor cells

**Fig. 6:**
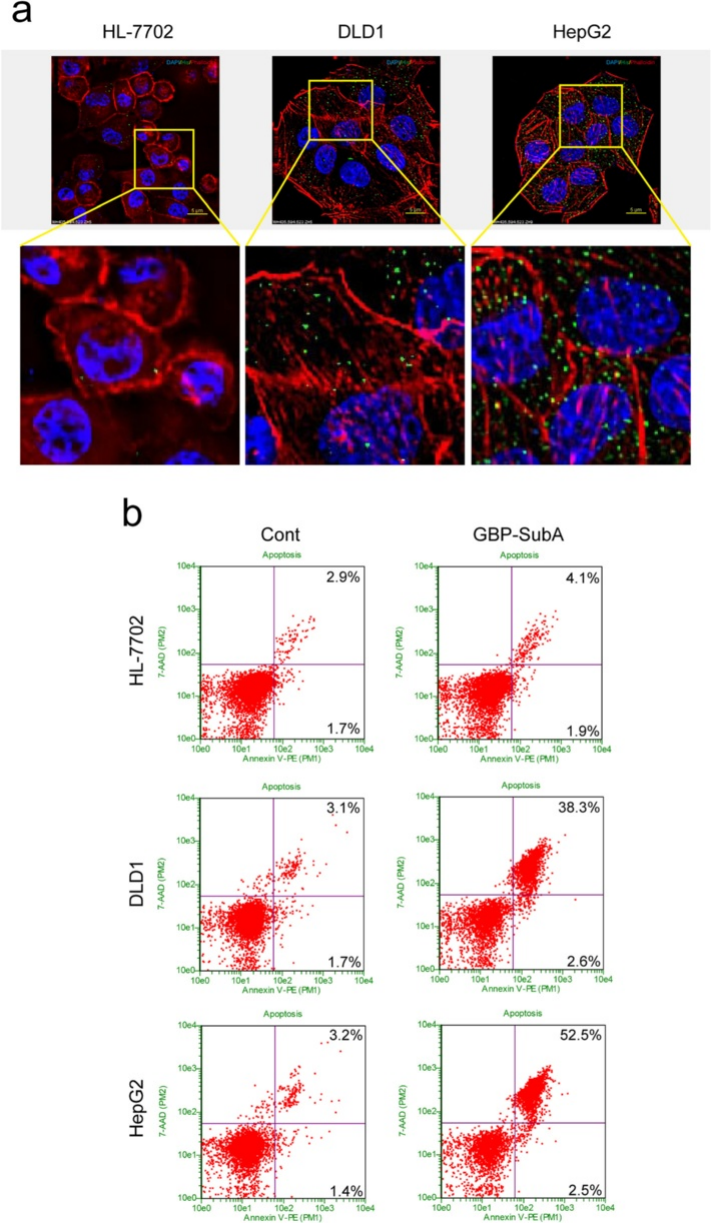
The apoptosis of tumor cells induced by GBP-SubA. **a**. HL-7702, DLD1 and HepG2 cells were incubated with 0.1 μg/mL GBP-SubA for 12 h. The entry of GBP-SubA to cells was analyzed by immunofluorescence. The GBP-SubA was stained by anti-His-tag primary antibody and FITC-conjugated secondary antibody, the actin was stained by Rhodamine-conjugated Phalloidin, and the nucleus was stained by DAPI. **b**. HL-7702, DLD1 and HepG2 cells were incubated with 0.1 μg/mL GBP-SubA for 48 h, the control was treated with PBS, and the apoptosis was analyzed by flow cytometry

To further detect the antitumor activity of recombinant GBP-SubA, we firstly examined the ability of GBP-SubA to enter cells at the concentration of 0.1 μg/mL by immunofluoresence staining. As described in Fig. 6a, GBP-SubA was detectable and predominantly presented in both DLD1 and HepG2 cells after incubated with GBP-SubA for 12 h, while there was no visible GBP-SubA in HL-7702 cells.

Next, the flow cytometric was applied to analyze the apoptosis-induction effect of GBP-SubA. As shown in Fig. 6b, GBP-SubA could induce 55 % HepG2 cells and 40.9 % DLD1 cells apoptosis (including early and late stage apoptosis), but the apoptosis rate was only 6 % in GBP-SubA-treated HL-7702 cells, which was close to the control (Fig. 6b). The results above implicated that GBP-SubA could specifically enter cancer cells and induce apoptosis.

### Verification of GBP-SubA bioactivity by blocking cell surface GRP78

**Fig. 7:**
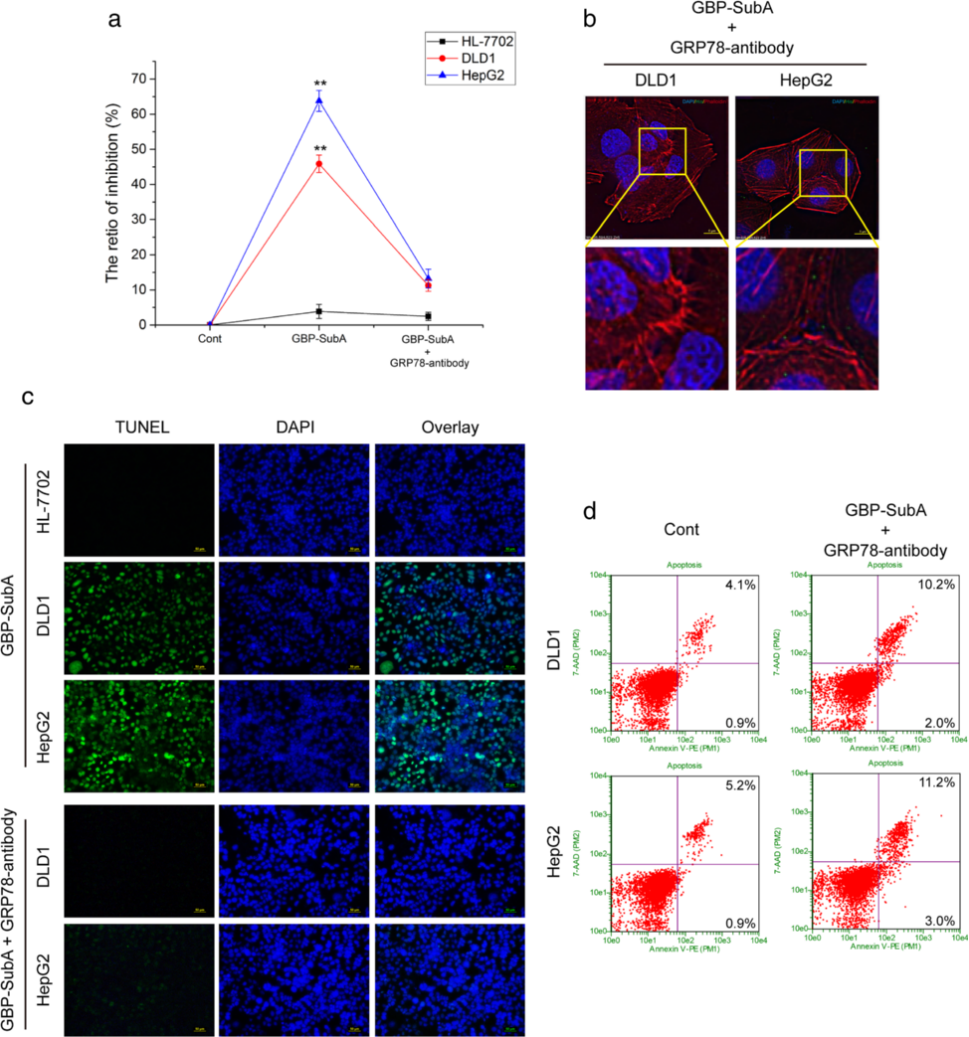
Verification of GBP-SubA bioactivity by blocking cell surface GRP78. **a**. After blocking cell surface GRP78, the cell inhibition ratios of GBP-SubA were determined by the MTT assay. HL-7702, DLD1 and HepG2 cells were incubated with or without anti-GRP78 antibody 1 h, and then 0.1 μg/mL GBP-SubA was added in the medium to incubate for another 48 h. *p < 0.05, **p < 0.01, compared with the control. **b**. After blocking cell surface GRP78, the cellular entry of GBP-SubA was detected by immunofluorescence. DLD1 and HepG2 cells were incubated with anti-GRP78 antibody 1 h, and 0.1 μg/mL GBP-SubA was then added in the medium to incubate for another 12 h. **c**. HL-7702, DLD1 and HepG2 cells were incubated with 0.1 μg/mL GBP-SubA for 48 h. For antibody blocking groups, the anti-GRP78 was added 1 h earlier. The nucleus morphology of apoptotic cells was observed by TUNEL and DAPI staining. **d**. After blocking cell surface GRP78, the apoptosis-induction effect of GBP-SubA was analyzed by flow cytometry. DLD1 and HepG2 cells were incubated with anti-GRP78 antibody 1 h, and 0.1 μg/mL GBP-SubA was then added in the medium to incubate for another 48 h. The control was without GBP-SubA and anti-GRP78 treatment

Theoretically, GBP-SubA could cleave the cell surface GRP78 at the COOH-terminal, which was the same as SubA [21]. Thus, to further verify the bioactivity of GBP-SubA on tumor cells, we blocked the tumor cell surface-GRP78 with antibody that against NH_2_-terminal of GRP78, and then detected the proliferation-inhibition effect, the cellular entry and the apoptosis-induction effect of GBP-SubA. The results of MTT assay showed that blocking cell surface-GRP78 reversed the proliferation-inhibition effect induced by GBP-SubA (Fig. 7a). Moreover, the amount of GBP-SubA getting into DLD1 and HepG2 cells was reduced significantly with the presence of anti-GRP78 antibody (Fig. 7b). Meanwhile, these cells were stained using TUNEL and 4, 6-diamidino-2-phenylindole dihydrochloride (DAPI) to show apoptotic morphology after incubation with GBP-SubA for 48 h. As illustrated in Fig. 7c, the HepG2 and DLD1 cells showed typical apoptotic nuclear changes, and the numbers of apoptotic bodies and TUNEL-positive nuclei in HepG2 were more than that in DLD1, and blocking surface-GRP78 could reverse the nuclear changes that were induced by GBP-SubA. However, GBP-SubA exerted very little effect on HL-7702 cells. Accordingly, the apoptosis rate of DLD1 and HepG2 fell to 12.2 % and 14.2 %, respectively (Fig. 7d). These results further demonstrated that GBP-SubA possessed the dual efficacy of targeting and killing tumor cells by against GRP78 only.

## Discussion

SubAB has been famous for high pathopoiesia since it was discovered. However, it is reported that nearly all toxins of bacterial or plant can be explored for tumor therapy through suitable modification, such as truncated diphtheria toxin (DT) or Shiga-like toxin (StxA), working through the inhibition of protein synthesis [22]. The ubiquitous receptor of B subunit handicaps the application of SubAB severely [23], so a tumor-specific assistant is necessary for the antitumor application of SubA. Epidermal growth factor (EGF) is the first ligand that is used to improve tumor cells specificity of SubA for the overexpression of epidermal growth factor receptor (EGFR) on tumor cell surface [24]. Nevertheless, EGFR not only presents on tumor cell surface, it also controls many different processes in normal tissues and cells, such as the differentiation and proliferation of astrocytes at late embryonic and neonatal stages of cortical development [25], the efficient differentiation of mesenchymal cells in the semilunar valves of the heart [26], as well as the cell proliferation and cell-cycle entry of hepatocytes following tissue injury [27], all of which limit the clinical applications of EGF-SubA.

GRP78 frequently locates on cell membranes of cancer cells but absent on those of normal cells, suggesting that tumor-targeted therapy via cell surface GRP78 is feasible. In this study, we chose GRP78 that is more specific distribution on tumor cell surface as the receptor, and GBP was employed as the ligand of GRP78, which is ligated to SubA to achieve targeting antitumor. The present data implied that the fusion protein GBP-SubA exhibited strong antitumor activities.

The ligand peptides GBP (WIFPWIQL) was designed to identify the cell surface GRP78 and perform further cell internalization. However, the specific interaction sites of GBP and GRP78 were still unknown. It has been reported that SubA is able to cleave cell surface GRP78 between the amino acid residues Leu^416^ and Leu^417^ and release the 28 kD COOH-terminal fragment of GRP78, which will abrogate the COOH-terminal domain signal transduction, whereas the NH_2_-terminal domain ligation is unaffected [21]. Logically, the GBP-SubA is also able to cleave the cell surface GRP78 as the same as SubA. If the interaction sites of GBP and GRP78 locate at the COOH-terminal fragment of GRP78, the fusion protein of GBP-SubA is impossible to display its function. Fortunately, our results clearly showed that the GBP-SubA was able to enter into the tumor cells, and the amount of GBP-SubA in cells was increased along with the enhanced cell surface GRP78, furthermore, blocking NH_2_-terminal of GRP78 with antibody could reduce GBP-SubA entry efficiency and reverse the proliferation-inhibition and the apoptosis-induction effects of GBP-SubA. Consequently, our results implied that the interaction sites of GBP and GRP78 may locate at the NH_2_-terminal of GRP78.

The main function of GBP (WIFPWIQL) in GBP-SubA is to identify the cell surface GRP78. The GBP composed of only eight amino acids, whether the tag for affinity purification blocked the function of GBP kept us concerned. It has been reported that the trypsin inhibitor fused with GBP and GST tag was able to bind to cell surface GRP78, which verified that the GST tag had no effect on the function of GBP [28]. We therefore speculated that His tag will have minimal effect on fusion protein, because 6× His is the minimum tag for protein purification. Based on this idea, we constructed GBP-SubA with pGEX-4T-1 vector and pET-28a vector, respectively. Our experiment further showed that the GBP-SubA with GST-tag was expressed as inclusion bodies in *E. coli* BL21 (DE3) cells, and the GBP-SubA with His-tag could not express in *E. coli* BL21 (DE3) cells (Additional file 1: Figure S1a and S1b). Moreover, we transfected the two recombinant plasmids into *E. coli* Rosetta (DE3), and found that both GBP-SubA of His-tagged and GST-tagged were able to express in Rosetta cells (Fig. 2a and Additional file 1: Figure S1c), but both of them expressed the fusion proteins as inclusion bodies. The GBP-SubA with His-tag was chosen to apply the denaturation and renaturation procedure due to its convenience of operations. As expected, through denaturation, purification and refolding, the His-GBP-SubA could enter into cells successfully, demonstrating that the His-tag had no effect on the function of GBP.

Aggregation of inclusion bodies is the major problem in the refolding procedure of denatured recombinant proteins. It had been reported that glycerol was able to increase the stability of proteins and aid proteins to refold [29]. In addition, cysteine residues could form inter- or intra-disulfide bonds that may lead to aggregation [30]. There are two cysteine residues in SubA that are going to form one disulfide bond (data from NCBI protein: 2IY9_A). Therefore, 5 % (v/v) glycerol and GSH/GSSG were added in the refolding buffer to facilitate the proper renaturation of GBP-SubA. During the refolding process, the renaturation rate is very important for the formation of native structure of protein. In order to slow down the rate of urea decrease in refolding buffer, the PBS was added dropwise at a rate of 100 mL/h. At last, 3.5 mg renatured GBP-SubA was obtained from 1 L culture.

## Conclusions

We successfully obtained the fusion protein GBP-SubA through expression, denaturation and renaturation. The GBP-SubA remained dual functions of both GBP and SubA. The obtained fusion protein was able to enter into cells by surface GRP78 and cleave intracellular GRP78, which induced tumor cells apoptosis. Therefore, the targeted suppression on tumors was achieved by GBP-SubA, which implied that GBP-SubA was suitable to development for antitumor drugs.

## Additional file

Additional file 1: Supplemental data. The sequence of primers for GBP-SubA and optimization of *E. coli* strain and vector of GBP-SubA expression. (DOC 710 kb)

## Abbreviations

Bip: Immunoglobulin heavy chain binding protein
DAPI: 4, 6-diamidino-2-phenylindole dihydrochloride
ER: Endoplasmic reticulum
GBP: GRP78 binding peptide
GRP78: Glucose-regulated protein 78
IPTG: Isopropyl-β-D-thiogalactopyranoside
PBS: Phosphate buffered saline
PVDF: Polyvinylidene difluoride
SubA: Subunit A of Subtilase cytotoxic
TRITC: Tetramethylrhodamineisothiocyanate
