# Supplementary material for: Design, purification and assessment of GRP78 binding peptide-linked Subunit A of Subtilase cytotoxic for targeting cancer cells
Source: BMC Biotechnol. 2016 Sep 1;16(1):65. doi: 10.1186/s12896-016-0294-5 (PMC5009487; doi:10.1186/s12896-016-0294-5)
Supplement: Additional file 1: Supplemental data. — The sequence of primers for GBP-SubA and optimization of E. coli strain and vector of GBP-SubA expression. (DOC 710 kb) [file 12896_2016_294_MOESM1_ESM.doc]

**Supplemental table 1. The sequence of primers for GBP-SubA**

| name | sequence |
| --- | --- |
| GBP-SubA | Forward：5′- CGCGGATCCTGGATTTTTCCGTGGATTCAGCTG-3′ |
|  | Reverse：5′- CCGCTCGAGTCATTACAGTTCTTCACTCATCCTT-3′ |

**Supplemental figure 1. Optimization of *E. coli* strain and vector of GBP-SubA expression.**

**
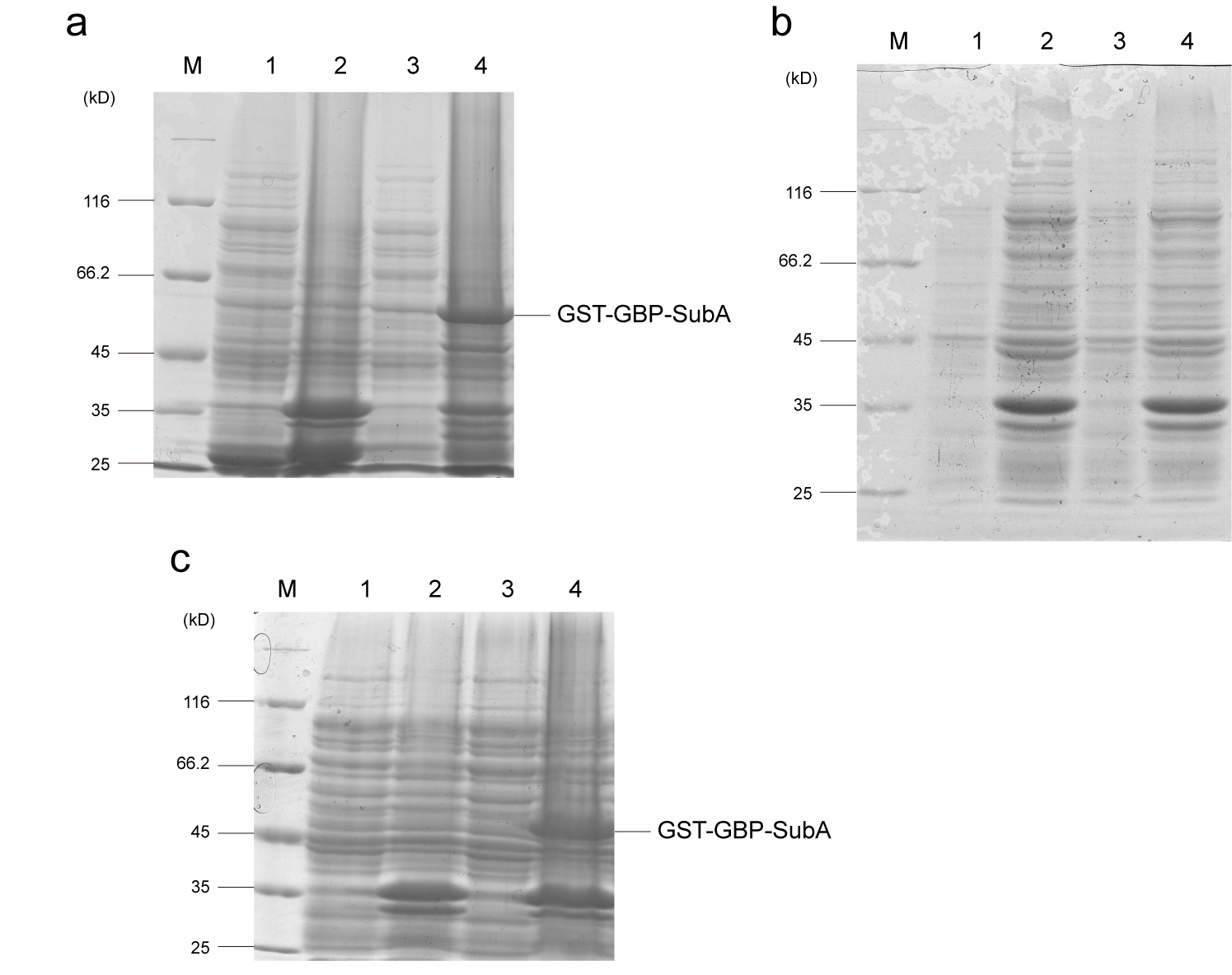
**

1. The expression of GBP-SubA with pGEX-4T-1 in *E. coli* strain BL21. Lane 1 and 2: soluble lysate fraction and insoluble lysate fraction of BL21 containing pGEX-4T-1; lane 3 and 4: soluble lysate fraction and insoluble lysate fraction of BL21 containing pGEX-4T-1-GBP-SubA.
2. The expression of GBP-SubA with pET-28a in *E. coli* strain BL21. Lane 1 and 2: soluble lysate fraction and insoluble lysate fraction of BL21 containing pET-28a; lane 3 and 4: soluble lysate fraction and insoluble lysate fraction of BL21 containing pET-28a-GBP-SubA.
3. The expression of GBP-SubA with pGEX-4T-1 in *E. coli* strain Rossate. Lane 1 and 2: soluble lysate fraction and insoluble lysate fraction of Rossate containing pGEX-4T-1-GBP-SubA without IPTG induction; lane 3 and 4: soluble lysate fraction and insoluble lysate fraction of Rossate containing pGEX-4T-1-GBP-SubA with IPTG induction.
